# The Genetic Legacy of the Pre-Colonial Period in Contemporary Bolivians

**DOI:** 10.1371/journal.pone.0058980

**Published:** 2013-03-20

**Authors:** Patricia Taboada-Echalar, Vanesa Álvarez-Iglesias, Tanja Heinz, Laura Vidal-Bralo, Alberto Gómez-Carballa, Laura Catelli, Jacobo Pardo-Seco, Ana Pastoriza, Ángel Carracedo, Antonio Torres-Balanza, Omar Rocabado, Carlos Vullo, Antonio Salas

**Affiliations:** 1 Unidade de Xenética, Instituto de Ciencias Forenses and Departamento de Anatomía Patolóxica e Ciencias Forenses, Facultade de Medicina, Universidade de Santiago de Compostela, Galicia, Spain; 2 Instituto de Investigaciones Forenses, Fiscalía General del Estado Plurinacional de Bolivia, La Paz, Bolivia; 3 Equipo Argentino de Antropología Forense, Córdoba, Argentina; 4 Laboratorio de Inmunogenética y Diagnóstico Molecular, Córdoba, Argentina; Institut de Biologia Evolutiva - Universitat Pompeu Fabra, Spain

## Abstract

Only a few genetic studies have been carried out to date in Bolivia. However, some of the most important (pre)historical enclaves of South America were located in these territories. Thus, the (sub)-Andean region of Bolivia was part of the Inca Empire, the largest state in Pre-Columbian America. We have genotyped the first hypervariable region (HVS-I) of 720 samples representing the main regions in Bolivia, and these data have been analyzed in the context of other pan-American samples (>19,000 HVS-I mtDNAs). Entire mtDNA genome sequencing was also undertaken on selected Native American lineages. Additionally, a panel of 46 Ancestry Informative Markers (AIMs) was genotyped in a sub-set of samples. The vast majority of the Bolivian mtDNAs (98.4%) were found to belong to the main Native American haplogroups (A: 14.3%, B: 52.6%, C: 21.9%, D: 9.6%), with little indication of sub-Saharan and/or European lineages; however, marked patterns of haplogroup frequencies between main regions exist (e.g. haplogroup B: Andean [71%], Sub-Andean [61%], Llanos [32%]). Analysis of entire genomes unraveled the phylogenetic characteristics of three Native haplogroups: the pan-American haplogroup B2b (originated ∼21.4 thousand years ago [kya]), A2ah (∼5.2 kya), and B2o (∼2.6 kya). The data suggest that B2b could have arisen in North California (an origin even in the north most region of the American continent cannot be disregarded), moved southward following the Pacific coastline and crossed Meso-America. Then, it most likely spread into South America following two routes: the Pacific path towards Peru and Bolivia (arriving here at about ∼15.2 kya), and the Amazonian route of Venezuela and Brazil southwards. In contrast to the mtDNA, Ancestry Informative Markers (AIMs) reveal a higher (although geographically variable) European introgression in Bolivians (25%). Bolivia shows a decreasing autosomal molecular diversity pattern along the longitudinal axis, from the Altiplano to the lowlands. Both autosomes and mtDNA revealed a low impact (1–2%) of a sub-Saharan component in Bolivians.

## Introduction

The Republic of Bolivia is located in central-south America. It is bordered by Peru to the West, Chile to the southwest, Paraguay and Argentina to the South, and Brazil to the North and East. Before the arrival of the Europeans, the Andean region of the country was an important part of the Inca Empire, the largest state in Pre-Columbian America, although the Inca civilization arose from the highlands of Peru in the early 13^th^ century. The Spaniards discovered the silver mines of Potosí in 1544 and soon began enslaving Natives as workers in the mines. The Spanish Empire conquered the region in the XVI century, and, during the colonial period, this territory was called Upper Peru. In the XVII century, the Spanish began bringing in African slaves in high numbers to help work in the mines, an institution that would last until abolition in 1826 (the independence of the country would arrive in 1825).

Nowadays, Bolivia is politically divided into nine departments and its geography varies from the high mountains in the Andes (West) to the eastern lowlands (Llanos), situated within the Amazon Basin.

About 10.2 million people live in Bolivia (Instituto Nacional de Estadística of Bolivia; INE; http://www.ine.gob.bo/). The country harbors a great cultural diversity. The number of individual languages known in Bolivia is 45; of those, 37 are living languages, one is a second language without mother-tongue speakers, and seven have no known speakers [1], [2]. The main language spoken today in Bolivia is Spanish, but there are also other important pre-Hispanic languages, such as Quechua (inherited from the Incan Empire; spoken by >1.6 million inhabitants), and Aymara (1.3 million inhabitants). The Quechua-speaking peoples inhabit mostly the (Sub)Andean valleys of Cochabamba and Chuquisaca and some mountain regions in Potosí and Oruro, while Aymara is mainly spoken in the high plateau (Altiplano) of the departments of La Paz, Oruro and Potosí (the area around Lake Titicaca). There are also other ethnic groups in the East, mainly living in the Llanos (including the Bolivian Amazon areas) e. g. Chiquitanos (>110,000 inhabitants), Guaraníes (>78,300 inhabitants; living mainly on the border with Paraguay), Moxeños (>76,000 inhabitants); these groups mainly occupy the departments of Santa Cruz, Beni, Pando and Tarija. The statistics vary slightly according to the different sources (see e.g. also [3])

The ethnic composition of Bolivia includes a great diversity of cultures but most indigenous peoples have assimilated a ‘mestizo’ culture. The Amerindian population accounts for approximately 55%; the remaining population is believed to be admixed with Europeans and Africans. There are more than 30 ethnic groups in Bolivia, the largest being Quechua- (about 1,500,000) and Aymara-speaking (25%). Several pseudo-ethnic terms are commonly used in Bolivia to self-designate their ancestries, such as ‘Mestizos’ (considered to be a mixture of Native Bolivians and Europeans), ‘Blancos’ (‘Whites’; considered to be descendants of Europeans or ‘Criollos’), ‘Afro-Bolivians’, Asians, and others (mainly Europeans from Germany, France, Italy, Portugal, and a minority of people coming from neighboring countries such as Argentina, Brazil, Chile, Colombia, etc.). The ‘Afro-Bolivians’ are descendants of African enslaved people, live mainly in the department of La Paz, and are concentrated in the provinces of Nor Yungas and Sud Yungas. Asian individuals are mainly Japanese (about 14,000) and Chinese (4,600).

Analysis of mtDNA variation has been used to explore demographic patterns in different regions of America, helping to unravel the origin of different ethnic groups, and the impact of modern migrations [4]–[9]. Some South American regions have received more attention in the literature than others; e.g. Argentina [10]–[12], Brazil [13], [14], Colombia [15]–[17], etc. However, limited genotyping efforts have been dedicated to Bolivians, and those have mainly focused on analysis of a few Native American Bolivians. Bert et al. [18] analyzed the mitochondrial DNA diversity in three different populations from the Llanos de Moxos (*n*  =  54) located in the lowlands of the Amazonian basin; the data indicated a higher genetic diversity in this locality than that observed in other American populations. In 2004, Dornelles et al. [19] analyzed a sample of Ayorea (*n*  =  91) individuals living in two Bolivian and one Paraguayan (neighboring) communities; the data suggested the effect of strong genetic drift in this population, significantly reducing the amount of variability. More recently, Corella et al. [20] analyzed several small samples from the Bolivian Piedmont, including Chimane, Moseten, Aymara and Quechua (*n*  =  100); the results suggest high genetic diversity in the area and high levels of inter-group variability. Afonso-Costa et al. [21] analyzed 111 individuals collected from La Paz, and the data was analyzed in a forensic context. Finally, Gayà-Vidal et al. [22] analyzed a sample of Aymaras and Quechuas from Bolivia (*n*  =  189); according to the authors, the data support a past common origin of the Altiplano populations in the ancient Aymara territory with independent although related histories with the Peruvian Quechuas.

Autosomal SNPs have been analyzed in only few studies. Galanter et al. [23] analyzed a panel of ancestry informative markers (AIMs) in a collection of pan-American samples, including a few from Bolivia. These authors found that most of the Bolivians have a main Native American component (ranging from 90% to 98%), with the exception of the Yungas province, showing a dominant African nature. Recently, Watkins et al. [24] carried out a genome-wide analysis of 28 Bolivians, and although the results agreed with Galanter et al. in that Bolivian genomes have predominant Native American features, they estimated a higher European component (12%) in these peoples.

The goal of the present study is to explore the variability of the Bolivian populations globally, including non-Native Bolivians from rural and urban populations, as well as those representing main departments and ecological regions (from the high mountains to the Llanos), and comparing them to previous studies that were carried out on a more local scale and to Native populations. Here, we have carried out the largest sampling of Bolivian populations to date (*n*  =  720). The data have been meta-analyzed jointly with previously available data from Bolivia and a large pan-American database. Whole genome sequencing was also carried out on selected mtDNAs in order to investigate Native American branches that have not been investigated before. In addition, AIMs have been genotyped in order to infer patterns of main continental ancestries that have contributed to the recent history of the country.

## Materials and Methods

### Sample collection

A total of 720 samples were recruited for the present study. They represent the three main regions of the country: a) Andean or Altiplano (*n*  =  240); b) Sub-Andean (*n*  =  204); and c) the Llanos (*n*  =  276) which to a large extent corresponds to the Bolivian Amazonian Basin. These samples were collected in the following Bolivian departments: Beni (*n*  =  102), Chuquisaca (*n*  =  89), Cochabamba (*n*  =  103), La Paz (*n*  =  253), Pando (*n*  =  90), and Santa Cruz (*n*  =  83). Birthplaces and other geographic information are given in **Table S1**. Additional samples (*n*  =  490) were collected from the literature: La Paz (*n*  =  110; [21]), Chimane (*n*  =  10), Moseten (*n*  =  10), Aymara (*n*  =  10) and Quechua (*n*  =  16) [20], Ayoreo (*n*  =  91; [19]), and Trinitario (*n*  =  12), Yuracare (*n*  =  15), Ignaciano (*n*  =  15), and Movima (*n*  =  12), all of which are around the small area of Llanos de Moxos [18], Aymara (*n*  =  96) and Quechua (*n*  =  93) [22].

A subset of the total samples (*n*  =  178), representing the departments of La Paz (*n*  =  105), and Chuquisaca (*n*  =  73), were genotyped for a panel of 46 ancestry informative markers (AIMs) in Heinz et al. [25]. Additional Bolivian samples selected from the total were genotyped *de novo* (*n*  =  420) and merged with those that were previously genotyped.

### Ethics statement

Written informed consent was obtained from all sample donors. Analysis of mtDNA sequences was approved by the institutional review boards of Santiago de Compostela (Spain). Moreover, the study conforms to the Spanish Law for Biomedical Research (Law 14/2007- 3 of July).

### DNA sequencing of the control region and entire genomes

Samples were PCR amplified and sequenced for HVS-I region, as described previously [26]. Entire genome sequencing was done as described in [27], [28].

We have followed the phylogenetic approach to scan the sequences as an *a posteriori* sequence quality control using the principles described in [29]–[31]. This filter was also applied to the data collected from the literature.

Nomenclature of mtDNA variants are referred against the revised Cambridge Reference sequence or rCRS [32], [33], and haplogroup nomenclature follows Phylotree Build 15 (www.phylotree.org; [34]); see also [35]. For the sake of inter-population comparisons and population summaries, we make the simplification that all A, B, C, and D haplotypes correspond to the Native American branches and not to the East Asian ones [36], given that specific mtDNA SNPs were not available. Therefore, along the text and figures, we used haplogroup labels according to the level of phylogenetic resolution used in the present study; e.g. B4 instead of B2; although it is most likely that all B4 belong to the Native American haplogroup A2. **Table S1** provides however the most accurate haplogroup classification according to the level of phylogenetic resolution obtained in the present study. Entire genomes generated in the present study are publicly accessible *via* GenBank with accession numbers KC503925 to KC503933.

### AIM-INDEL genotyping

Bolivian samples were genotyped for 46 INDEL markers [37] in a multiplex PCR amplification and capillary electrophoresis process. Each AIM-Indelplex PCR amplification was performed with 5 µl 2x Quiagen Multiplex PCR Master Mix, 10x Primer Mix, and 0.5 µl DNA (concentration between 0.5 – 5 ng/µl) in a final volume of 10 µl. PCR thermocycling conditions were: initial temperature of 95°C for 15 min; 28 cycles at 94°C for 30 sec, 60°C for 90 sec, and 72°C for 60 sec; final step at 72°C for 60 min. Following amplification, 0.8 µl PCR product was added to 11.5 µl Hi-Di Formamide (Applied Biosystems) and 0.3 µl Liz-500 Size Standard (Applied Biosystems). DNA fragments were separated according to size using a 3130 Genetic Analyzer (Applied Biosystems) and were analyzed with GeneMapper (Applied Biosystems).

### Statistical analysis and molecular dating

Haplotype (*H*) and nucleotide (*π*) diversities, and mean number of pairwise differences (*M*) were calculated using DnaSP v.5 software [38]. Arlequin 3.5.1.2 [39] was used to compute AMOVA (Analysis of Molecular Variance) and the significance of the covariance components associated with different levels of genetic structure were tested on haplotype frequencies applying a non-parametric permutation procedure. Population pairwise *F_ST_* values, between/within population average nucleotide pairwise differences, and Nei’s inter-population distances, were also computed using Arlequin 3.5.1.2 [39].

Diversity indices, phylogeographic inferences and inter-population comparisons were carried out using the sequence range 16090 to 16365, since this is the most commonly reported segment in the literature. Problematic variation located around 16189, which was usually associated with length heteroplasmy, e.g., 16182C or 16183C, was ignored.

Fisher’s exact test and Pearson’s chi-square test were undertaken using the R package (http://www.r-project.org/); a significant nominal value of α  =  0.05 was considered.

Maximum parsimony trees were built for the complete genomes obtained in the present study and those collected from the literature. For each cluster, the time to the most recent common ancestor (TMRCA) was calculated by computing the averaged distance (ρ) of all the haplotypes in a clade to the respective root haplotype. Heuristic estimates of the standard error (σ) were calculated from an estimate of the genealogy, as done in [40]. Hotspot mutations (16182C, 16183C and 16519) were excluded from calculations (as usual). The corrected evolutionary rate proposed by Soares et al. [41] was used to convert mutational distances into years. The TMRCA values obtained in the present study could be slightly over-estimated as indirectly suggested by estimates obtained in the literature regarding the entrance of the first Paleo-Indians into the American continent [4], [5], [9]. Therefore, the TMRCA values obtained here should be validated using a larger number of entire genomes.

Analysis of population structure was undertaken using the software STRUCTURE 2.3.4 [42]–[44]. Both, burn-in and Markov Chain Monte Carlo (MCMC) repetition were set to a length of 100,000. Parameters were selected as indicated in Heinz et al. [25]. Furthermore, the reference samples obtained from the Human Genome Diversity Cell Line Panel, HGCP-CEPH [45], were used to assist in clustering; this panel constitutes a collection of 556 reference samples representing four main continents: Africa (*n*  =  105), Europe (*n*  =  158), America (*n*  =  64), and East Asia (*n*  =  229). Each run was repeated five times from *K*  =  2 to *K*  =  7. Structure Harvester (http://taylor0.biology.ucla.edu/structureHarvester/) was used to estimate optimal *K* values. CLUMPP 1.1.2 [46] and Distruct 1.1 [47] were used to prepare data for visualization as bar plot representations. R 2.13.0 [48], together with the SNPassoc package [49], was used to run two- and three-dimensional Principal Component Analysis (PCA). We compared and evaluated the results obtained from both approaches.

Snipper [50] (http://mathgene.usc.es/snipper/) was used to make four-way predictions of ancestral origin (Africa, Europe, East Asia, and Native Americans) of Bolivian profiles. SNP data collected from HapMap populations were also used as training sets (http://hapmap.ncbi.nlm.nih.gov). Prediction was based on maximum likelihood.

## Results

### Mitochondrial DNA molecular diversity in Bolivia

Several diversity indices have been computed considering different hierarchical levels (**Table 1**). When analyzed by main regions, all seemed to show similar diversity values; with the Department of La Paz harboring the lowest haplotype diversity, and Chuquisaca being the region with the lowest nucleotide diversity. The highest haplotype diversity was found in the North (Pando) but the highest nucleotide diversity is observed in the South (Santa Cruz). Therefore, there is no obvious correlation between departments and molecular diversity as measured by way of statistical summary indices. When examining the diversity by rural and urban populations, it was observed that rural populations harbor higher nucleotide and haplotype diversity than urban populations.

**Table 1:** Diversity indices in Bolivian mtDNAs and main American and African regions.

Populations groups	Reference	n	*k*	*S*	*H*	*π*	M
**Department**							
Beni	Present study	102	63	71	0.982±0.002	0.02470±0.00130	6.818
Chuquisaca	Present study	89	57	59	0.971±0.009	0.01588±0.00099	5.718
Cochabamba	Present study	103	71	77	0.981±0.007	0.01855±0.00086	6.677
La Paz	Present study	253	121	81	0.960±0.007	0.01864±0.00086	5.106
Pando	Present study	90	54	60	0.983±0.006	0.01977±0.00068	7.117
Santa Cruz	Present study	83	49	46	0.978±0.006	0.02618±0.00097	7.226
All Bolivia	Present study	720	306	134	0.976±0.002	0.02237±0.00041	6.130
**Rural *vs*. Urban**							
Rural	Present study	189	108	92	0.980±0.004	0.02440±0.00069	6.709
Urban	Present study	531	240	121	0.975±0.003	0.02225±0.00049	6.098
**Regions**							
Andean	Present study	240	116	78	0.961±0.007	0.01856±0.00088	5.086
Sub-Andean	Present study	204	118	90	0.973±0.006	0.02215±0.00083	6.092
Llanos	Present study	276	141	100	0.982±0.003	0.02458±0.00059	6.759
**Native groups**							
Aymara-speakers (Beni)	[20]	10	5	9	0.667±0.163	0.00499±0.00251	1.800
Moseten (Beni)	[20]	10	8	18	0.956±0.059	0.01761±0.00209	6.356
Quechua-speakers (Beni)	[20]	16	7	17	0.692±0.124	0.01094±0.00358	3.950
Chimane (Beni)	[20]	10	8	16	0.933±0.077	0.01422±0.00212	5.133
Aymara-speakers	[22]	96	39	48	0.956±0.009	0.01645±0.00142	4.523
Ayoreo	[19]	91	8	10	0.473±0.061	0.00626±0.00104	2.260
Ignaciano	[18]	15	11	23	0.933±0.054	0.01773±0.00237	6.400
Movina	[18]	12	8	12	0.894±0.078	0.00814±0.00174	2.940
Quechua-speakers	[22]	93	40	48	0.946±0.012	0.01997±0.00118	5.471
Trinitario	[18]	12	11	22	0.985±0.040	0.01884±0.00212	6.803
Yuracare	[18]	15	11	22	0.952±0.040	0.01852±0.00151	6.686
All Native		380	114	84	0.946±0.006	0.02201±0.00043	6.008

The indices were computed using the common segment of the HVS-I region from position 16090 to 16365.

NOTE: *n*  =  sample size; *K*  =  number of different haplotypes; *S*  =  number of segregating sites; *H*  =  haplotype diversity; *π*  =  nucleotide diversity; *M*  =  average number of pairwise differences (mismatch observed mean).

The most apparent geographic pattern was found when examining molecular diversity values by main ecological region. The Andean followed by the Sub-Andean regions had substantial lower diversity values than the Llanos. Therefore, the diversity was found to increase longitudinally, from the high mountains to the lowlands of the Llanos.

Diversity was particularly low in some ethnic groups compared to general urban and rural populations. For instance, the Ayoreo (from Bolivia and Paraguay) and the Aymara had extremely low diversity values compared to the average values observed in Native Bolivians and Bolivians in general (**Table 1**), a fact that is most likely due to strong founder effect in the case of the Ayoreo [19], while in the Aymara discussed by Corella et al. [20] it was most likely due to the small sample size analyzed (the values were compared with those obtained with the Aymara population from Gayá-Vidal et al. [22] using a larger sample size). Our sample from general rural and urban Beni showed significantly higher diversity values than those observed from other Native American groups from the same department (e.g. the Piedmont populations of Moseten, Chimane, etc [20], the Llanos populations analyzed in [18]; see **Table 1**).

There are only few haplotypes in Bolivia that appears more than two or three times in the whole dataset, again indicating the high mtDNA diversity of the country (**Figure S1 and Figure S2**). When evaluating the differentiation between departments, it was again observed that comparisons of other departments with Santa Cruz were the ones that displayed the highest *F_ST_* values (**Figure S3**). The influence of this department is also indirectly observed when studying *F_ST_* values between the main ecological regions, indicating that the comparisons between Llanos and the other two areas showed the largest values (**Figure S4**).

Finally, **Figure S5 and Figure S6** show the expected (virtual) heterozygosity in the main departments and in the main regions, respectively; the dispersion of values indicated the existence of substantial mtDNA diversity between departments.

### Phylogeography

**Figure 1:**
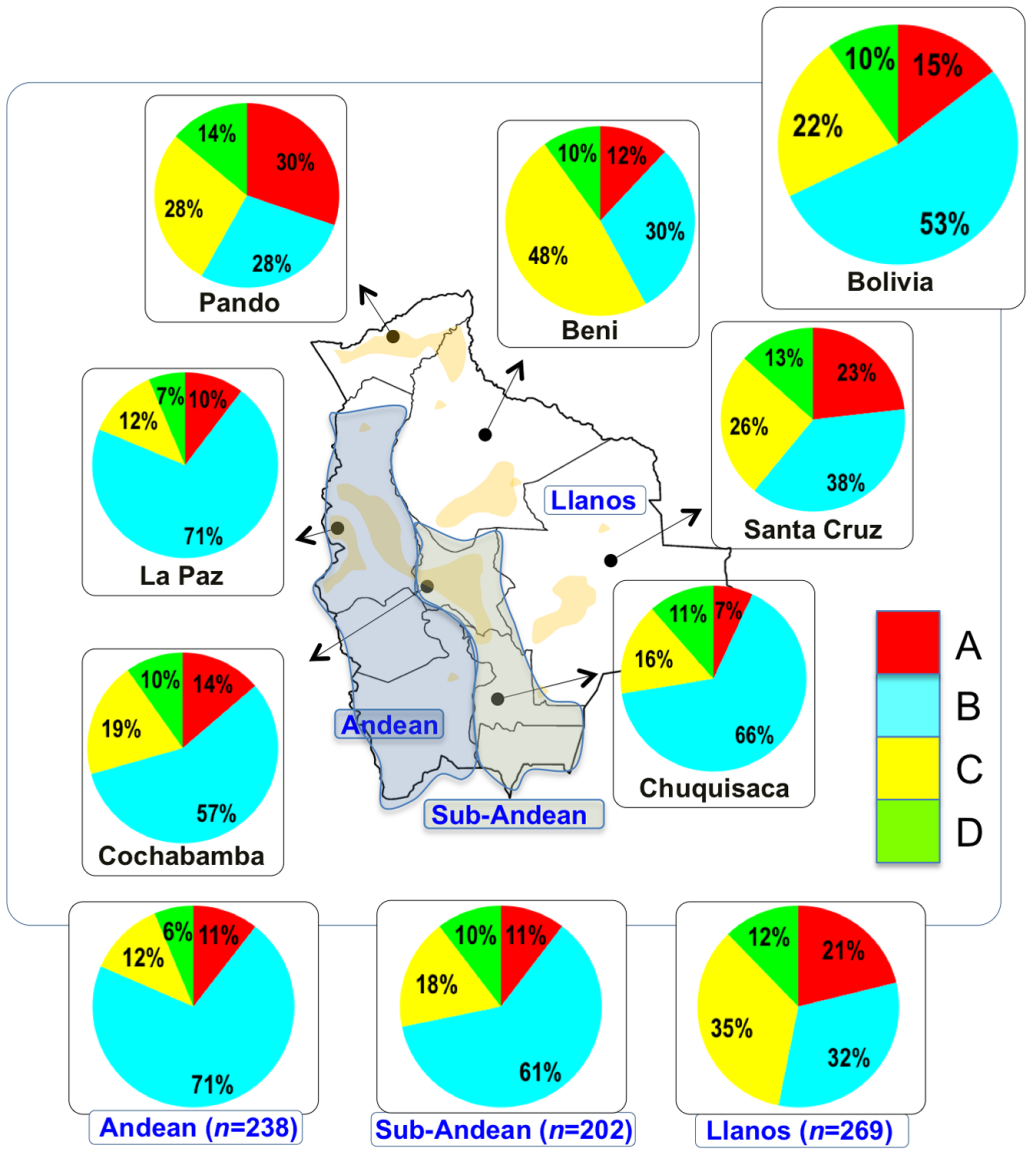
Map of Bolivia showing the location of the samples collected in the present study. The pie charts represent the distribution of basal Native American haplogroup frequencies in the region.

The mtDNA Native American component predominates in the Bolivian population (98.4%); most of the variation can be classified into one of main Native American haplogroups, (A: 14.3%, B: 52.6%, C: 21.9%, D: 9.6%), **Figure 1**.

There were remarkable differences in haplogroup frequencies between the main Bolivian departments (**Figure 1**). For instance, the department of La Paz had the following haplogroup composition, A: 10%, B: 71%, C: 12%, and D: 7%, which was in sharp contrast with the composition observed in the department of e.g. Beni, A: 12%, B: 30%, C: 48%, and D: 10%. The haplogroup distribution found by Afonso-Costa et al. [21] in a smaller sample from La Paz was slightly different to that found in the Bolivians from La Paz studied here, but both samples agreed regarding the high proportion of haplogroup B in this area. This geographic pattern mirrors in reality the location of the departments at different altitudes; thus, the most important differences are observed between Andean and Sub-Andean populations *versus* Llanos. For instance, haplogroup B is the predominant haplogroup in the Altiplano (71%), which decreases to 61% in Sub-Andean populations and 32% in Llanos (**Figure 1**).

As expected, Bolivia shares more haplotypes with South America than with Meso and North America (**Table 2** **; Table S2**), although these values have to be interpreted with care due to the different sample sizes. Note however that the largest sample size is in North America, but Bolivia only shares 18% of the haplotypes with this region but 49% with other South American populations (**Table 2**). It is also worth mentioning that 48 haplotypes are shared between the three American regions; therefore, of the 68 haplotypes shared with North America, most of them are also shared with South America (**Table 2** **, Table S2**). A large amount of haplotypes has been only observed in Bolivia (250 out of 383 different haplotypes; 65%).

**Table 2:** Shared haplotypes between Bolivia and other American regions.

	*N*	SH with Bolivia	H	H_1_
North America	7551	68	1875	1085
Meso-America	2792	68	925	612
South-America	5727	187	1587	1009
Bolivia	1127	383	383	250

Only the segment 16024 to 16385 was considered for comparison. **N**  =  sample size; **SH**  =  shared haplotypes; **H**  =  number of different haplotypes; **H_1_**  =  number of unique haplotypes per region.

There are eight mtDNAs that most likely belong to haplogroup C1d (all carry the characteristic transition 16051); there is another mtDNA that could be allocated to the northern South American cluster C1d2a, which does not carry transition 16091 but carries transition 16209 instead. C1d has been reported as one of the founding Paleo-Indian mtDNA lineages of the American continent [5] and is therefore found along the double continent. As has been previously described [5], C1d did not necessarily follow a main Pacific coastal route once it reached South America; this would explain why this lineage appears more in the Llanos than in the Andean region.

The five mtDNAs belonging to D4h3a found in our Bolivian samples most likely arrived at about the same time as C1d but probably followed a different route. According to [4], D4h3a appeared to spread along the Pacific coast. In agreement with this hypothesis, the five D4h3a Bolivians were observed in the Andean region, and three out of the five D4h3a lineages belong to the Peruvian sub-clade D4h3a3.

According to expectations, no members of the Paleo-Indian founder X2a, which has a North American distribution, have been found in Bolivians. Also, in good agreement with Bodner et al. [9], we did not find any members belonging to southern cone lineages D1j and D1g, favoring the hypothesis that these two haplogroups crossed the Andes at lower latitudes (Chile towards Argentina), where they were most likely incubated.

Very recently, de Saint Pierre et al. [51] proposed two new lineages, B2i2 and C1b13, which are thought to have originated in the Southern Cone, and are closely associated with the Paleo-Mapuche people in Chile and Argentina. Members of these two lineages have only been sporadically found outside of Chile and Argentina. Here, we only found one member of C1b13, and according to the hypothesis formulated by de Saint Pierre et al. [51], this mtDNA could have arrived in Bolivia from the South instead of from the West.

Some Bolivian mtDNAs are particularly interesting given that they appear at relatively high frequencies in this region and neighboring areas but are absent in the rest of the continent. For instance, the motif 16189 16217 16290 appears six times within haplogroup B4 in different regions from Bolivia; it also appeared in four Andean Peruvian Quechua individuals [52]. The motif 16173 16192 16223 16298 16325 16327 16346 (haplogroup C) appears twelve times in Bolivia, and all of them in the Llanos. Curiously, this motif was found also in another individual from La Paz in [21] and another one from Chaco, in the North of Argentina, close to the Bolivian Llanos [11].

Haplogroup L (L referring to all mtDNA branches excluding macro-haplogroups M and N) mtDNAs represent the sub-Saharan exclusively maternal genetic component of the country, which in this case only accounts for 1% of the total mtDNA pool (**Table S1**). The proportion of African recent ancestry in Bolivia is very low compared to other South American countries that were more influenced by the African slave trade [16], [17], [53]. There are only seven different haplotypes belonging to some of the typical sub-Saharan sub-clades. Individual #BNI19 belongs to L0a1b2, which is spread in all of sub-Saharan Africa as well as in America, but carries a distinctive transition (within L0a1) at position 16271 (which gets an score of nine in the mutation list of Soares et al. [41]). The L1b (#BNI75) and L1c1a1 (#LaPaz467) profiles most likely come from the West-Central African region. The L1c3b1a and the L3f1b4a profiles appear in Cabinda, Angola and Mozambique [54]–[57], but also in West-Central, in Gabon [58]. As expected [53], [59], the most likely geographic origin of the African Bolivian profiles is west-central Africa, but also encompasses the Southwest and the Southeast.

It is also curious that the European mtDNA component represents less than 1% of the Bolivian population. With the level of resolution of the HVS-I segment, it is not possible to assign European lineages to a particular region in Europe; the four sequences observed here belong to haplogroups H1af, K and X1. An Asian origin for the profile C16104T C16223T cannot be disregarded given that two exact haplotype matches appeared in China [60] but any in Europe (in our in-house database of more than 26700 HVS-I profiles).

### Entire genomes from Native American Bolivians

In order to investigate the Native American lineages in our Bolivian samples further, we selected those showing a distinctive pattern from the control region data. Thus, we chose two mtDNAs carrying the tandem variants T16097C A16098G on top of the sequence motif for haplogroup A2, and a group of six mtDNAs all carrying transition G16145A on top of the B2 basal motif (**Table S3**). Entire genome sequencing revealed some interesting phylogeographic features of these mtDNAs.

**Figure 2:**
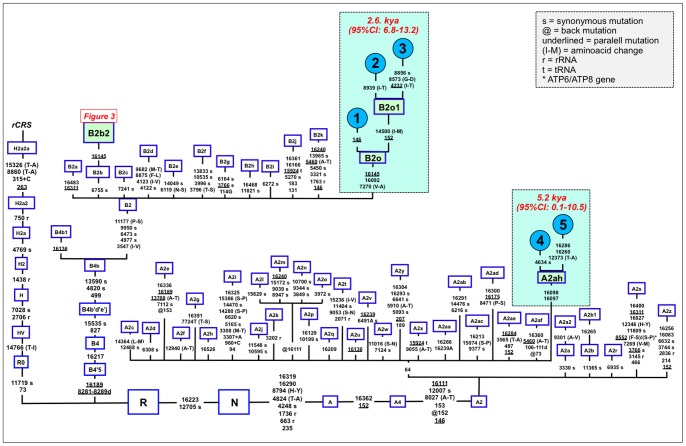
Maximum parsimony tree of the main branches characterizing haplogroups A2 and B2, indicating the new branches generated in the present study, namely, A2ah and B2o. The mutations are displayed along the branches: numbering is according to the rCRS [71]: a mutation alone indicates a transition while an upper case letters as suffix (A, C, G, T) indicates a transversion; “+” and “d” refer to insertions and deletions, respectively; “s” refers to a synonymous variant while non-synonymous variants are indicated by way of the amino acid change; a star indicates a variant located in the *ATP6* or *ATP8* gene; a prefix @ indicates a back mutation; and underlined positions indicate parallel mutations. Sample ID (see Table S3) for the entire genomes generated in the present study are as follows: #1: COBIJA577, #2: COBIJA604, #3: STACRUZ261, #4: BENI86, and #5: STACRUZ221.

The tandem transitions T16097C A16098G conform *per se* a very distinctive motif that is found very rarely in mtDNA databases. Within haplogroup A2, this motif alone (without any other control or coding region variant) defines a novel branch of the Native American phylogeny, here named as A2ah (**Figure 2**). The two genomes show variability within this clade and, given its very restrictive location within Bolivia, this most likely point to an origin in Bolivia or the surrounding territories. We further investigated A2ah in public databases of entire mtDNA genomes. We found just one entire genome (JQ702082) that carries the transition A16098G on top of the A2 motif but it differs substantially from the two genomes found in Bolivia (apart from lacking the transition T16097C); therefore, its inclusion within A2ah should be considered to be very dubious. According to the entire genome sequences available, it can be said that haplogroup A2ah originated about 5,200 thousand years ago (kya) (although with a large 95%CI: 0.1–10.5). We additionally investigated the phylogeographic characteristics of these lineages by looking at the abundant amount of data available in the literature on control region segments. We only detected nine mtDNAs matching the motif of A2ah. Four out of ten were observed in the data reported by Behar et al. [61] but geographic information was not available. Two other HVS-I mtDNAs were found in the ‘Hispanic’ North American subset of the SWGDAM database [62]; three other A2ah mtDNAs were observed in South America, two among the Brazilian samples of Alves-Silva et al. [13], and one in the Toba from Gran Chaco (North Argentina) [63]. In Bolivia, only four A2ah members were found (0.5%), all of which were observed in the Llanos (three in Santa Cruz and one in the Beni department). In general, the HVS-I data suggest that this lineage could have originated in Bolivia or some place in Central South America. The two members observed in the ‘Hispanic’ samples from the SWGDAM could perfectly represent recent immigrations into USA. The motif T16097C A16098G was also observed in Mainland Scotland (Western Islands; Isla of Skye) and two other samples in [61], but none of the mtDNAs carried the variation defining haplogroup A2.

**Figure 3:**
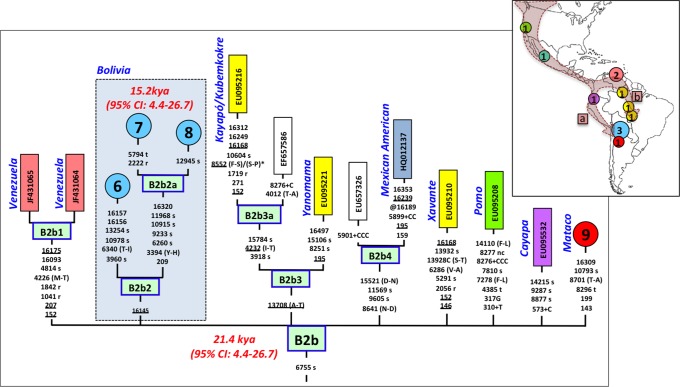
Maximum parsimony tree of haplogroup B2b. The map on the top right inset indicates the geographic location of the B2b genomes represented in the tree; circles are proportional to the sample size. The arrows indicate the two tentative migration routes (a and b in the map) of this lineage (see main text) along the American continent. References for entire genomes are as follows (see **Table S1** and **S3**): #6: BENI90, #7: STACRUZ258, and #8: COBIJA110, #9 Mco-10 (present study, **Table S1** and **S3**). For more information, see the legend of **Figure 2**.

A phylogenetic conflict exists when trying to reconstruct the most parsimonious tree of the six B2 mtDNAs carrying transition G16145A. Three of the genomes (#6, #7 and #8 in **Figure 3**) carried the synonymous transition G6755A, while three other genomes (#1, #2 and #3) lacked G6755A but carried the non-synonymous transitions T7270C and T16092C instead (**Figure 2**). Given that G16145A seems to have a much higher mutation rate than G6755A (22 *versus* 2 mutational hits in Soares et al. [41], and 24 *versus* 5 in Phylotree; respectively), we decided to resolved the phylogeny as shown in **Figure 2** and **Figure 3**.

Thus, the motif T7270C T16092C G16145A defines a new branch of the Native American phylogeny named here as B2o, which is represented by three Bolivian genomes (**Figure 2**). No other entire genome was observed in public resources belonging to B2o. Dating the phylogeny of B2o (**Figure 2**) indicates that this haplogroup originated about 2.6 kya (95%CI: 6.8–13.2). Searching control region databases, we observed only two HVS-I candidate B2o sequences: one was observed in San Martin de Pangoa in Perú, a small town on the eastern slope of the Andes inhabited by Quechua and Nmatsiguenga people [64], while the other B2o sequence was observed in Guam (an island located in the western Pacific Ocean, territory of USA). The latter, however, carries the motif T16189C T16217C A16247G C16261T, which is very common in the Micronesia, Australia, etc. [65], [66] and points to a different clade of the worldwide phylogeny, haplogroup B4a. In Bolivia, we found seven B2o representatives (1%), most of them in the Llanos (five in Pando and one in Santa Cruz departments), and one in a Sub-Andean locality of La Paz. Therefore, altogether the data suggest that this lineage has also originated in Bolivia or some nearby region, most likely located in the Andes.

The entire genomes of the three Bolivian mtDNAs carrying G16145A but lacking T16092C, all share the transition G6755A. By searching the databases on entire genomes for the transition G6755A, we observed 10 additional mtDNAs belonging to this branch. In reality, this clade had already been described in the recent literature [6], [7] and was named B2b. However, its internal variation was never analyzed in detail. **Figure 3** shows the phylogeny of B2b and the geographical location where the entire genomes were observed. Geographic and/or ethnic affiliation was available for eleven entire genomes (including the Bolivian ones): one Pomo (North California, USA) [8], one Mexican American [67], two Venezuelans from Pueblo Llano [68], one Yanomama (Brazil, Venezuela) [8], one Cayapa (Equador) [6], one Kayapó/Kubemkokre (South Amazonia, Brazil) [8], one Xavante (Brazil) [8], and three Bolivians (present study). We additionally detected one entire genome in our DNA databank in a Mataco Native from North Argentina, which was added to the phylogeny of **Figure 3** (#9). According to the phylogeny of B2b in **Figure 3**, B2b appeared about 21.4 kya (95% CI: 4.4–26.7). The Bolivian sub-clade B2b2 is much younger, approximately 15.2 kya (95%CI 4.4–26.7). In the large HVS-I database, we only observed 14 B2b candidates, almost overlapping the territories represented by the entire genomes. In Bolivia, we counted seven B2b candidates: one Andean (La Paz), four Sub-Andean (Cochabamba, La Paz and Chuquisaca), and two in the Llanos (Beni and Santa Cruz).

### AMOVA analysis of mtDNA profiles

AMOVA was undertaken considering different hierarchical levels; by Department, Rural *vs*. Urban, and Andean *vs*. Sub-Andean *vs*. Llanos. As expected, the within-population variation accounted for most of the variance (ranging from 93–100%; **Table 3**), independently of the population sub-division employed.

**Table 3:** AMOVA computed based on haplotype pairwise differences of Bolivian populations (significant tests: 20,022 permutations; adjusted *P*-value<0.0000).

	WithinPopulations	AmongPopulations
Rural *vs*. Urban	99.85	0.15
Andean *vs.* Sub-Andean *vs*. Llanos	93.15	6.85
Departments	93.44	6.56
Provinces	93.60	6.40

AMOVA carried out by departments and ecological region provided the highest values of among-population variance (ranging from 10.48 to 11.58% of the total variance). The values are similar given the correlation that exists between these two classification criteria. The rural *vs*. urban division indicates virtually no correlation among group subdivision. The analysis carried out by province did not increase the among-population variance, indicating again that the main factor influencing within-population variation was the different altitudes in the country.

### Continental ancestry in Bolivians

A panel of 46 AIMS was genotyped in Bolivians in order to infer their main continental ancestry. Data from main continental reference samples (Africa, East Asia, Europe and America) were used as classification sets.

**Figure 4:**
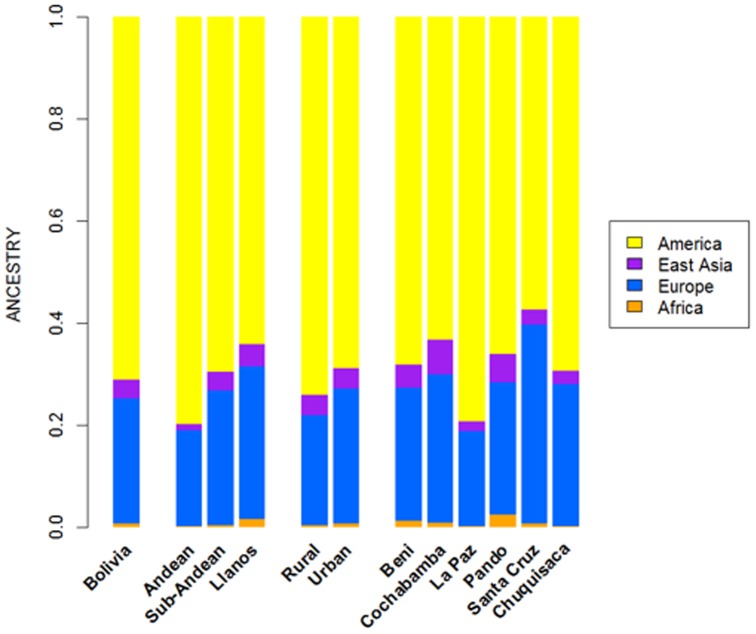
Average continental ancestry of Bolivians analyzed using a panel of 46 AIMs. These values are obtained from the STRUCTURE analysis and the optimal value *K*  =  4.

**Figure 5:**
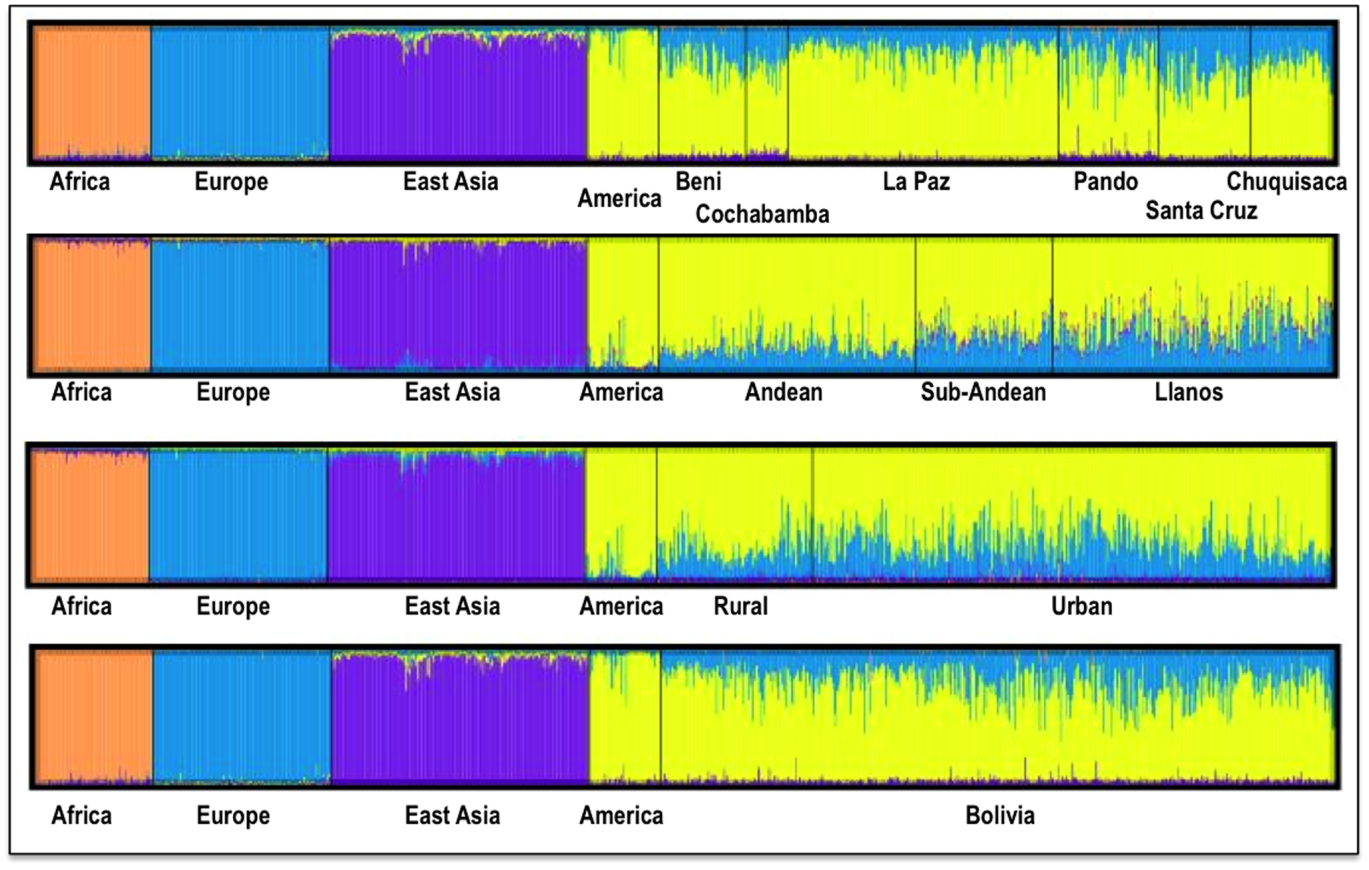
STRUCTURE analysis of Bolivians based on the 46 AIMs panel genotyped in the present study. Each bar plot represent analysis using different grouping schemes for the Bolivian samples. Only the results for the optimal *K*  =  4 are represented (see complete analysis in **Text S1**).

Analysis of ancestry was carried out using STRUCTURE. **Figure 4** summarizes the average continental ancestries observed in Bolivians under different grouping schemes, while **Figure 5** shows the STRUCTURE bar-plots. The analysis showed that, on average, 71% of the component in the total Bolivian sample is Native American, followed by 25% of European ancestry. When examining the ancestry by departments, La Paz was the region that showed the highest Native American ancestry (79%) (**Figure 4** and **Figure 5** **)**, and, therefore, the lowest European component (19%). On the other side is the department of Santa Cruz, with 57% of Native American ancestry and 39% European. The African component was very low in all of the departments, showing the highest value in the department of Pando (2.5%), in the North. When examining by ecological regions, it was also evident that the Native American component is higher in the mountainous West (80%), and decreases progressively eastward: sub-Andean (70%) and Llanos (64%). The differences are less apparent when examining rural *vs.* urban areas (74% and 69% of Native American component, respectively).

The STRUCTURE bar-plot of **Figure 5** indicates the ancestral membership for each individual in the source population and the Bolivian samples. It clearly shows that most of the individuals have a Native American ancestry, with only a few exceptions. The high European component of Santa Cruz compared to other departments is also evident in this bar-plot, and is also evident in the Andeans. When looking at the most extreme values, we found that there are only a few that have European ancestry above 50% in most of the departments. It is, however, rare to see individuals with high African membership; the highest values correspond to 23% in one individual from La Paz and another one from Pando (17%).

The membership of individuals into the East Asian cluster is significantly higher in some individuals, as is also evident in the STRUCTURE plots. However, this perhaps mirrors the fact that the separation between the Native American component and the East Asian one is not perfect. The overlap between Native Americans and East Asians can be observed more clearly in the PCA analysis (see below). This fact has been already observed in Pereira et al. [37]. Thus, most of the East Asian component would be captured by the Native American cluster in cases using a three-group structure analysis (Native American, African, and Europe).

**Figure 6:**
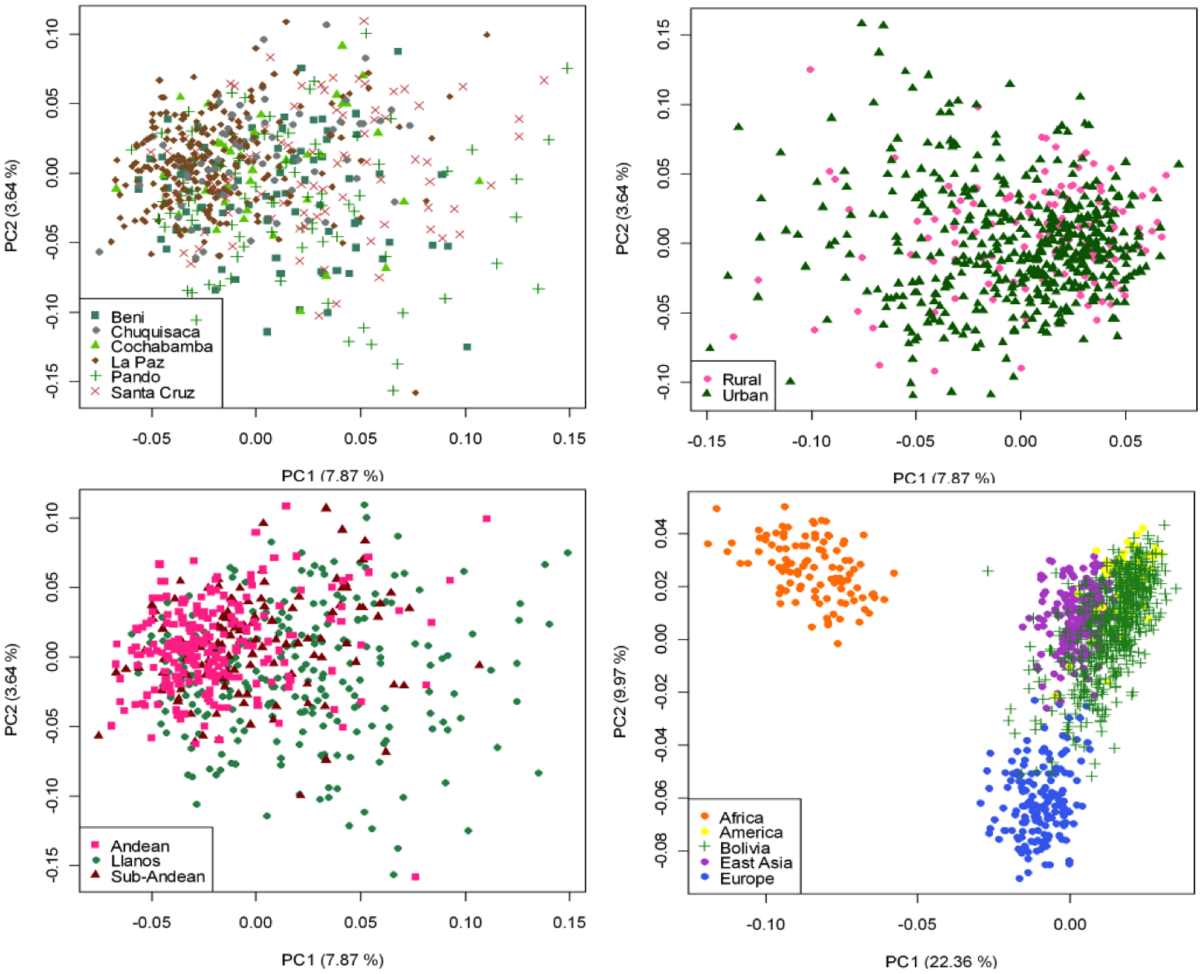
PCA of Bolivian profiles based on the 46-AIMs panel genotyped in the present study. Each figure shows different grouping schemes mainly aiming to show the within-Bolivian diversity.

The PCA agrees well with the results observed in STRUCTURE. The Bolivian profiles group mainly with the Native American ones from HapMap, with only some samples showing a projection towards the European cluster (**Figure 6**). The minor African component detected in the STRUCTURE analysis seems not to be relevant in the PCA; only one sample show some affinity to the African cluster; it is in fact the same sample with a 23% African ancestry in STRUCTURE. The PCA carried out by departments (**Text S1**) showed that the department of La Paz (Andes) was more tightly grouped than the profiles from other departments; in the other pole are the departments of Santa Cruz and Pando, showing more dispersed patterns (Llanos). This west-east pattern is more evident when observing the PCA by main regions (**Figure 6**): the Andean profiles are more tightly grouped in one pole of the plot, while the profiles from Llanos are in the other pole and are more dispersed, with the sub-Andean profiles occupying an intermediate position between East and West. This dispersion is also mirrored when examining the standard deviation (SD) of the Native American membership values in the main regions: (i) Andean: mean  =  79.7%, SD  =  0.086%; (II) sub-Andean: mean  =  69.6%, SD  =  0.09; and (iii) Llanos: mean 64.2%, SD  =  0.137% (**Table S4**); given that the department of La Paz is basically an Andean department, it also shows the lowest SD (0.85%).

**Figure 7:**
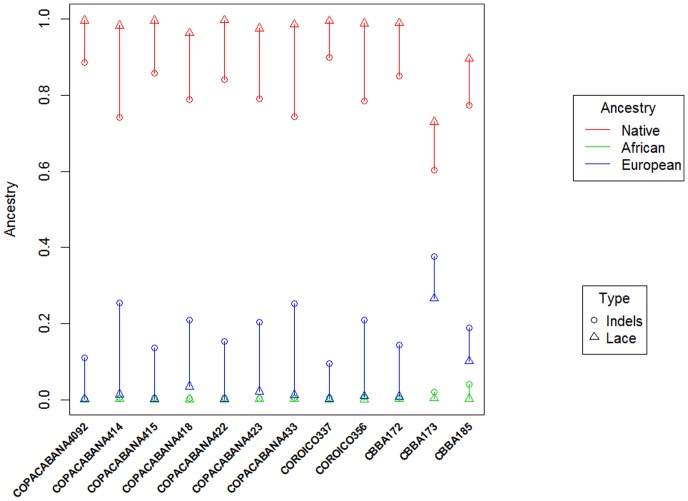
Percentages of main continental ancestry inferred using different AIMs: the 46-Indels panel analyzed in the present study *versus* the 446-SNP LACE panel analyzed in [23]. The information is only available for 12 individuals from Bolivia. The vertical bars only highlight the difference of ancestry observed using different panels for each individual

Finally, measuring ancestry using AIMS depends on many factors, such as the use of different panels of SNPs (not only different SNPs, but also different numbers of them), different classification test samples, sample sizes, etc. Twelve of the samples genotyped in the present study for the 46-indel panel were also genotyped in Galanter et al. [23], thereby offering a good opportunity to investigate the ability of different AIM panels to estimate percentages of continental ancestry. As shown in **Figure 7**, the ancestry memberships obtained using Indels lead to a decreased Native American ancestry (and proportional increased European ancestry) compared to the estimates obtained using the LACE panel (on average, the difference is about 15%). Given the way in which the LACE panel was designed (in order to balance the informative content of the different AIMS included in the panel), it seems logical to believe that the Indels panel would tend to over-estimate the proportion of European ancestry in Bolivians. Note, however, that the genome-wide study of Watkins et al. [24] carried out in few Bolivians estimated a higher European component (12%) compared to the LACE. Further genome-wide studies on a larger Bolivian sample will allow better estimates of continental ancestries to be obtained.

## Discussion

Bolivia shows a mainly Native American mtDNA component (98%). Only 1.5% of the profiles have Sub-Saharan mtDNA ancestry. The impact of Europeans in the Bolivian mtDNA pool is minimal (0.5%), which also contrasts with other South American locations [11], [27]. Although the Native American mtDNA component predominates in the country, there is a highly diverse and geographic stratification in the country. In a large geographical scale, the largest difference corresponds to Llanos *vs.* the Andean and Sub-Andean regions. The political definition of the departments overlap quite well with the main latitudes observed in the country, which would explain the correspondence observed when carrying out different statistical analysis. Molecular diversity is extremely low in some indigenous group compared to urban and other rural populations, suggesting the existence of important episodes of isolation and genetic drift in Native communities. AMOVA carried out at Bolivian populations to different hierarchical levels allowed a better understanding of the spatial geographic patterns of variability in large regions. The results agree that most of the variation accounts within populations, but that the major differentiation occurs between the Andes and the Llanos. In addition, some mtDNA phylogeographic features indicate the presence of common lineages between different Andean regions in Peru and Bolivia, most likely testifying for the common demographic past during the Inca’s Empire period. This continuity in the Andean region was also observed in the Aymaras and Quechuas analyzed by Gayá-Vidal et al. [22].

By way of sequencing the entire genome of nine mtDNAs (eight from Bolivia and one from a northern Argentinean Native Mataco), and compiling a large amount of data from the literature, we could shed light on three Native American lineages: A2ah, B2o and B2b. The three haplogroups are rare in Bolivia (ranging from 0.5 and 1%) and are very rare continentally. The three clades show significant diversity within the Bolivian country, indicating that they most likely evolved locally after their arrival from other neighboring regions. The clade for which there are more data available (literature and present study) is B2b. This haplogroup most likely arose in North California; however, as suggested by its TMCRA (∼21.4 kya; see also [67]) and its phylogeographic characteristics, it could have been originated even in the north most region of the American continent, constituting a new minor Paleo-Indian founder. The data also indicate that B2b traveled South, most likely following the Pacific coastline (the main route followed by the First Americans) [4], [5], [9], [69]. It probably entered the South American sub-continent following two different paths: (i) travelling further south following the Pacific side, then reaching Equador, Peru, and Bolivia; and (ii) following firstly an eastwards direction, and secondly southwards crossing the Amazon basin in Venezuela and then Brazil (or firstly bordering the Atlantic coast southwards). At the very least, these lineages arrived in northern Argentina, but it is likely that further sampling will probably detect B2b in the most southern edge of the continent. The data indicate that the Bolivian sub-lineage B2b2 arrived in this region about 15,000 years ago.

Ancestry analyses carried out on a panel of AIMs indicated that Bolivians have a substantial European ancestry (although a possibility exists that this proportion could be over-estimated; see above), which varies substantially between departments, thus indicating a differential regional impact of the European Colonialism in Bolivia. The African component is very low (in agreement with the mtDNA) as compared to other South American populations, such as the Caribbean coast [17], [70], Colombia [16], and Brazil [13], but is comparable to others such as Argentina [10], [11], [27]. It must be taken into account that the present study did not include samples from the Yungas, a small province located within the department of La Paz that seems to concentrate the main African component in Bolivians. The African component of the Yungas people is not only inferred from their distinguishable African cultural and biological features, but also from the genetic inferences made using panels of AIMs analyzed in a small group of people from this region [23].

The present study represents the largest and most comprehensive analysis of genetic variation investigated to date in rural and urban Bolivians from the point of view of mtDNA and autosomal data. The results indicated that even those Bolivians that did not self-identify as belonging to a particular Native America ethnic group still preserved a main Native American genetic character in their genomes. Deep analyses of selected mtDNA genomes also indicate the presence of lineages that appears to be autochthonous to these peoples; provided that these lineages have not been identified in large American databases. The Native American component of Bolivians is significantly higher when observing the mtDNA instead of the autosomes. This is a common feature in other South and Central American regions (e.g. Argentina [10], Panama [2])where the maternal Native American component has been found to be much better preserved in the maternal specific genome, a fact that is generally explained by the higher proportion of European males arriving to America than females.

## Supporting Information

Figure S1 **Frequency of different haplotypes (alleles in figure) by department.**
(TIFF)

Figure S2 **Frequency of different haplotypes (alleles in figure) by ecological region.**
(TIFF)

Figure S3 **Fst values between departments.**
(TIFF)

Figure S4 **Fst values between ecological regions.**
(TIFF)

Figure S5 **Expected (virtual) heterozygosity by departments.**
(TIFF)

Figure S6 **Expected (virtual) heterozygosity by main ecological regions.**
(TIFF)

Table S1 **Mitochondrial DNA sequencing data for the Bolivian samples analyzed in the present study.** Note that for a large proportion of sequences the range is generally larger than what it is usually considered to be HVS-I and HVS-II.
(XLSX)

Table S2 **Shared haplotypes between Bolivia and other American locations.**
(XLSX)

Table S3 **Information on entire genome sequences obtained in the present study and collected from the literature and GenBank.**
(XLSX)

Table S4 **Continental ancestry values (standard deviations in brackets) obtained using STRUCTURE for the Bolivian samples averaged through five different iterations (see Material and Methods for more information).** The minimum and the maximum values are also given.
(XLSX)

Text S1 **Additional PCA and structure analyses carried out in the Bolivian samples based on 46 AIMs.**
(DOCX)
