# Supplementary figures and images for: The Genetic Legacy of the Pre-Colonial Period in Contemporary Bolivians
Source: PLoS One. 2013 Mar 20;8(3):e58980. doi: 10.1371/journal.pone.0058980 (PMC3604014; doi:10.1371/journal.pone.0058980)

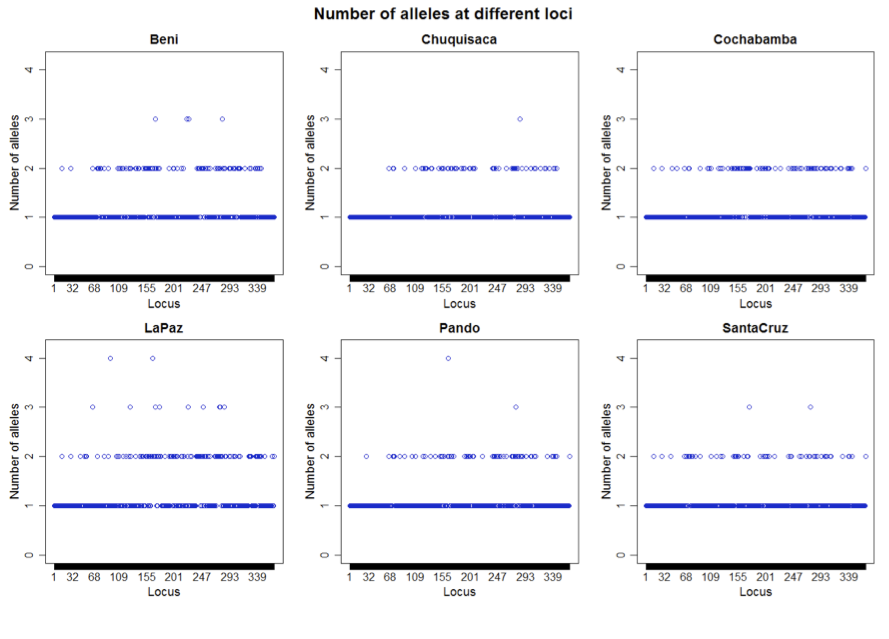

Supplement: Figure S1 — Frequency of different haplotypes (alleles in figure) by department. (TIFF) [file pone.0058980.s001.tif]

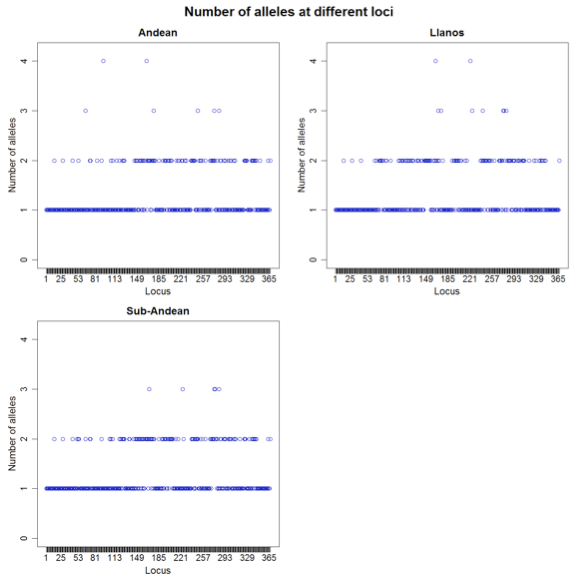

Supplement: Figure S2 — Frequency of different haplotypes (alleles in figure) by ecological region. (TIFF) [file pone.0058980.s002.tif]

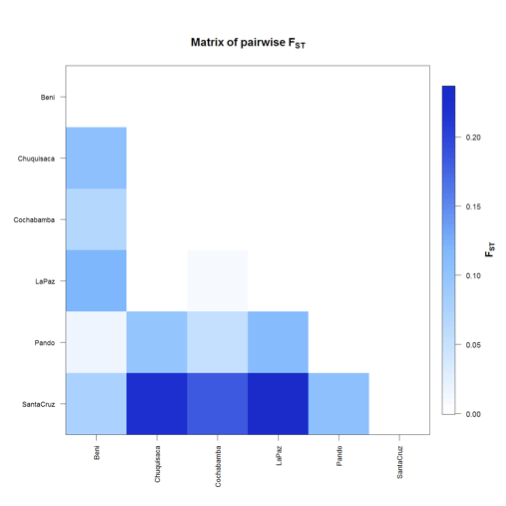

Supplement: Figure S3 — Fst values between departments. (TIFF) [file pone.0058980.s003.tif]

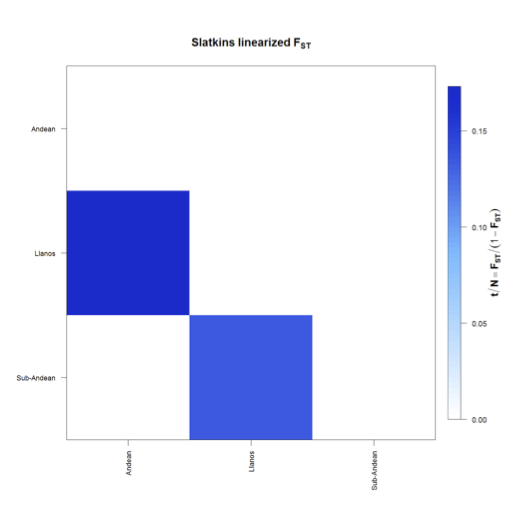

Supplement: Figure S4 — Fst values between ecological regions. (TIFF) [file pone.0058980.s004.tif]

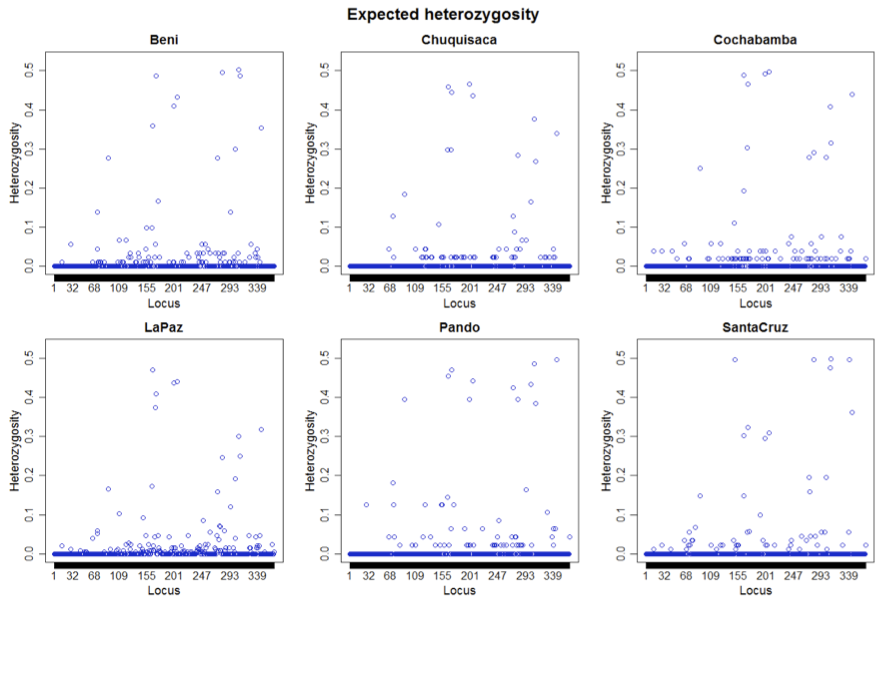

Supplement: Figure S5 — Expected (virtual) heterozygosity by departments. (TIFF) [file pone.0058980.s005.tif]

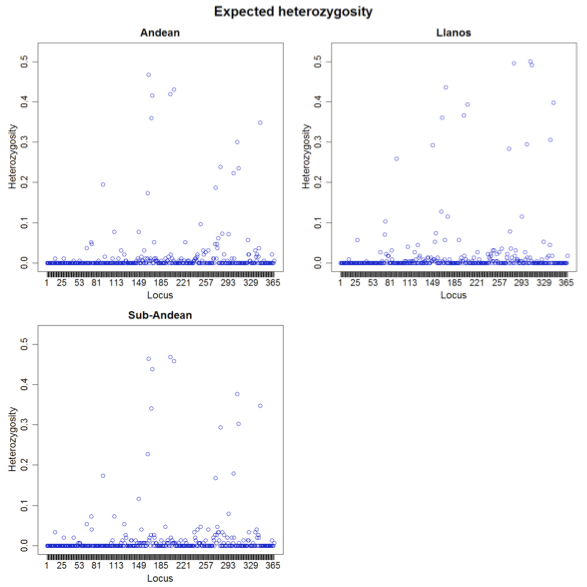

Supplement: Figure S6 — Expected (virtual) heterozygosity by main ecological regions. (TIFF) [file pone.0058980.s006.tif]
