# Supplementary material for: The Genetic Legacy of the Pre-Colonial Period in Contemporary Bolivians
Source: PLoS One. 2013 Mar 20;8(3):e58980. doi: 10.1371/journal.pone.0058980 (PMC3604014; doi:10.1371/journal.pone.0058980)
Supplement: Text S1 — Additional PCA and structure analyses carried out in the Bolivian samples based on 46 AIMs. (DOCX) [file pone.0058980.s011.docx]

**TEXT S1.**

This document summarizes analysis that were carried out on the 46 AIMs by provinces, and main Bolivian regions.

**Figure S1. PCA analysis on the department of Beni**

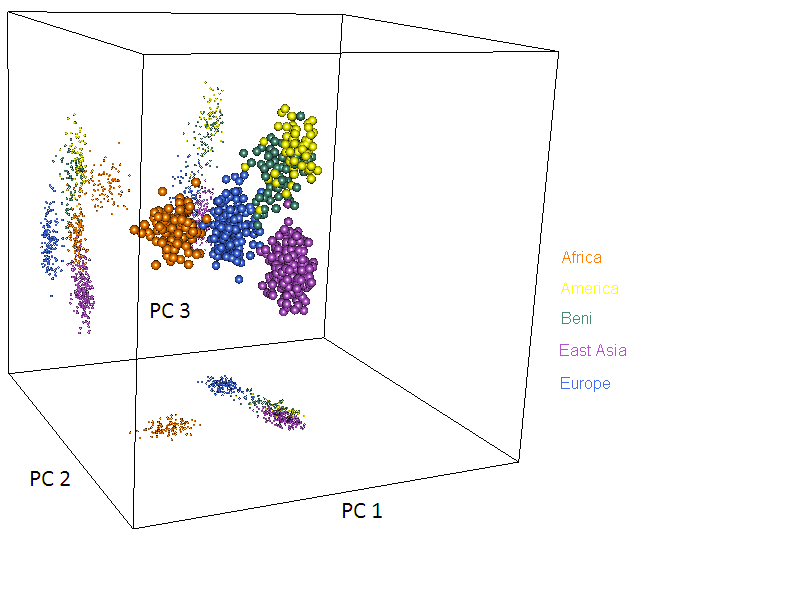


**Figure S2. PCA analysis on the department of Cochabamba**

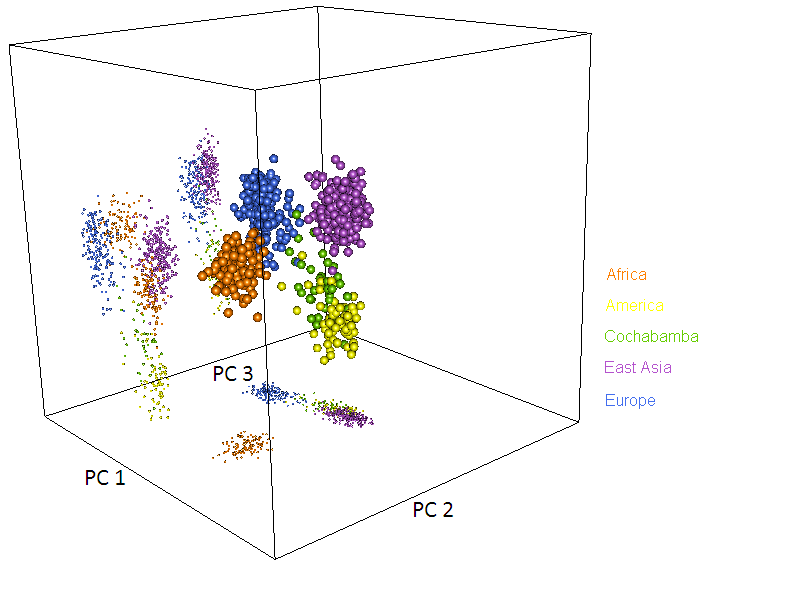


**Figure S3. PCA analysis on the department of Chuquisaca**

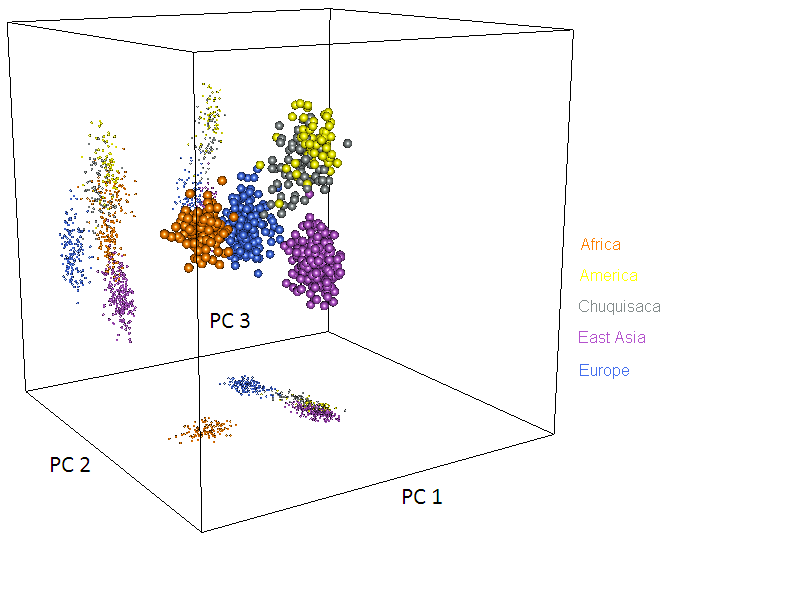


**Figure S4. PCA analysis on the department of La Paz**

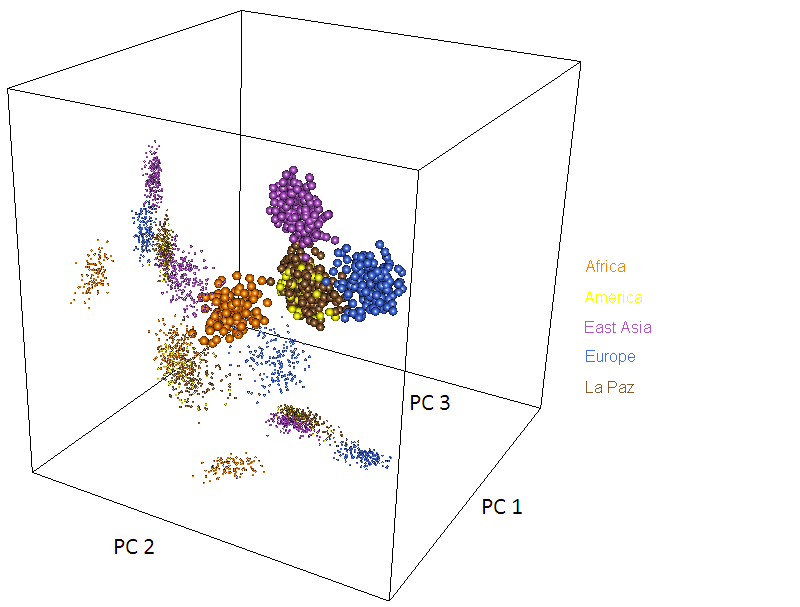


**Figure S5. PCA analysis on the department of Pando**

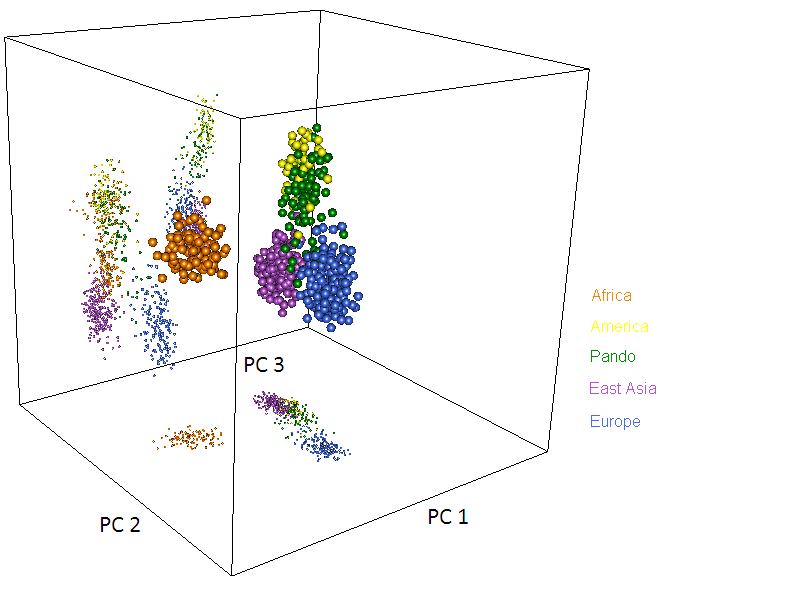


**Figure S6. PCA analysis on the department of Santa Cruz**

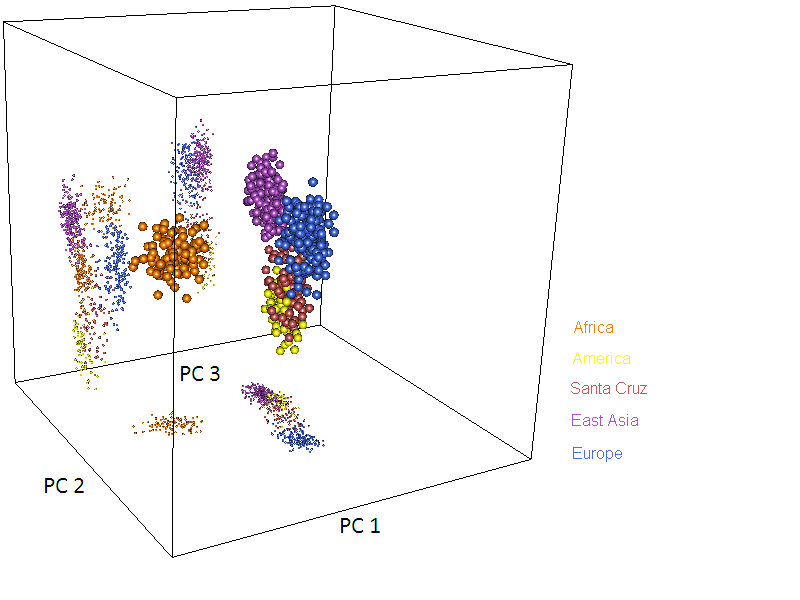


**Figure S7. Structure analysis carried out by departments**

**Figure S8. Structure analysis carried out by main regions**

**Figure S9. Structure analysis carried out in rural and urban locations**

**Figure S9. Structure analysis carried out in the whole Bolivian sample**
